# Goggle-free swimming as autonomous water competence from the perspective of breath control on execution of a given distance

**DOI:** 10.1038/s41598-024-69996-y

**Published:** 2024-08-13

**Authors:** Marek Rejman, Daria Rudnik, Robert Keig Stallman

**Affiliations:** 1 Department of Swimming, Faculty of Physical Education and Sport, Wroclaw University of Health and Sport Sciences, Wroclaw, Poland; 2 Department of Physical Performance, Norwegian School of Sport Sciences (Retired), Oslo, Norway

**Keywords:** Aquatic education, Drowning prevention, Breath control, Goggles, Adolescents, Environmental social sciences, Health care

## Abstract

This study aimed to examine the ability of adolescents to maintain breathing rhythm while swimming with and without goggles, in the context of pedagogical interventions for implementation of water competence skills, rather than simply teaching swimming technique (strokes). 25 females and 25 males, 12–13 years old, swam the front crawl both with goggles and without goggles. Distance covered and the ability to maintain breathing rhythm were evaluated by experts. For both girls and boys, the lack of goggles reduced the breath control. The boys in contrast to the girls, could "swim" (cover a distance) but did not have the “competence” to swim effectively/safely—with breathing rhythm—regardless of the goggle factor. Goggle-free swimming as an autonomous component of water competence is highly recommended in elementary swimming education. The following elements for pedagogical intervention in the area of water competence development are proposed: (1) the formatting of breath control on the basis of the student's preferred, simplest form of swimming (not strokes); (2) the a priori treatment of swimming goggles as an unnecessary teaching aid; (3) the gender differences in area of both adaptation in visual perception (the goggles factor) and motor control (breath control factor) should be considered.

## Introduction

Given that more than half of water-related fatalities are persons under the age of 25^1^, aquatic education for children and adolescents is particularly important. Scientific activity in the field of improving the effectiveness of swimming education towards drowning prevention dates back to the early nineteenth century^2^ and demonstrated the need to extend the skill set of a 'good swimmer' to include the utilitarian dimensions of 'scientific' and 'imaginative' swimming. The contemporary concept of water readiness and aquatic literacy^3^ encompasses all activities in and around the water, including skills that go beyond the displacement of the body in this environment. This is complemented by the requirement to provide learners with individual experiences and interactions with the aquatic environment^4^. Thus, the notion of water competence began to take shape with the contribution of Stallman et al.,^5^ who emphasized the didactic value of the integration of knowledge, attitudes and behaviors that focus on safety in and around water, with aquatic skills.

Water competence refers to the range of cognitive, affective, and psychomotor experiences associated with all behavior in the aquatic environment. This includes the ability to successfully apply these competencies under unfavorable internal conditions, such as emotions and fatigue, and external conditions, such as temperature, waves, water currents, and clothing^6,7^. The development of these competencies is attained through the acquisition of knowledge and behavioral experiences, resulting in the evolution of skills and also involves adopting certain universal values, positive attitudes, and rational behavior in and around water, with human life as a paramount concern^5,7^.

Acquiring water competence is a dynamic process that depends on the interaction between the individual, the aquatic environment, and the individual's actions in that environment^4^. Although swimming ability is a fundamental component of water competence, it is not the only one^3,8^. It is therefore important to avoid uncritical belief in one's swimming ability, as this can lead to dangerous situations^9–11^. Overestimating one's ability is a common cause of fatal accidents in the water^1,12,13^. Therefore, the goal of pedagogical intervention should not be limited to teaching swimming techniques (strokes). Instead, it should raise awareness to the insufficiency of a focus on strokes in the context of safety in and on water^8^. The learn to swim process only makes pedagogical sense if it leads to acquiring aquatic competencies and their further improvement with the intention of preventing drowning^4,6,11,13^.

From a pedagogical perspective, the definition of aquatic literacy is also proposed. Here it is understood as a pedagogical process that serves as a preliminary stage for the learner to be prepared to acquire water competence in the terms of skills. At the same time, by arousing only positive emotions, desirable attitudes and behaviors are created to provide the learner with the natural, individual need to acquire complete water competence. As with the acquisition of literacy, the development of aquatic literacy provides the foundation for further education in water competence. Learning and mastering of these basic skills in this process provides a real sense of confidence and opens up a range of opportunities for safe participation in a water-related activities. In this way, children and young people are introduced to the ecological model of physical activity^14^ and it is hypothesized that they will be encouraged to develop a lifelong need for aquatic activity, together with positive attitudes and behaviors in and around water. The definition in question appears to have a clear implementation in educational practice. This is evidenced by the link between “fundamental swimming skills and water competencies”^15^.

Due to its specific nature (mainly its density), water limits the human ability to move. According to the theory of Newell^14^, and later, Sallis et al.^16^ and Xu et al.^17^, it was assumed that important substrates of these limitations in the processes of aquatic literacy and learning to swim are visual impairment and difficulty in maintaining breathing rhythm. Therefore, this study explores the proposition that swimming skills without goggles and the ability to breathe rhythmically while swimming (inhaling above the surface and exhaling below the surface) should be considered as essential water competencies.

The use of goggles in contemporary teaching of swimming, remains controversial. Swimming goggles are seen by many researchers and practitioners as having both 'enabling' and 'disabling' constraints due to visual impairment in the water^18^. The use of goggles may have important benefits for non-swimmers by making swimming easier^19^. The most crucial aspect in this context is reducing learners' fear (or other negative emotional attitudes like skepticism, suspicion, etc.) of water, which is one of the biggest barriers to learning how to swim^20,21^ and the strongest predictor of poor swimming skills^22^. With goggles, vision is not obstructed, thus underwater visibility may reduce initial reluctance to facial immersion^19^. Calm submerging of the face makes it easier to feel buoyancy and also helps to obtain and keep a horizontal body position. The complex coordination of arms legs and breathing control needed for other swimming activities may then be simplified^23^. Swimming with goggles, is also sometimes viewed as an aid in teaching aquatic literacy, including enhancing confidence^19^. In these cases goggles might make the learning process more comfortable. Here, the question arises as to whether the learner's habit of swimming with goggles, which eliminates the physical and psychological discomfort of eye contact with the water, will affect their swimming skills in a way that could pose a risk if they are suddenly in the water without goggles. In this sense, the process of learning to swim that begins without goggles is not treated as a source of constraint but is considered essential. Considering also the research of Moran and Stanley^11^ the ability to swim without goggles appears to be a crucial skill for increasing water competence and ensuring safety in emergency situations. As such, the ability to swim without goggles should be treated as an autonomous water competence.

As noted above, goggles may facilitate breath control including rhythmic breathing which is a prerequisite for the ability to achieve hand-breath coordination^23^. Rhythmic breathing is a recognized determinant of swimming ability^24,25^ and effective breathing is a key factor in swimming economy^5^, in terms of minimizing the effects of increasing fatigue^26^. Rhythmic breathing allows for continuous swimming, which increases self-esteem, self-confidence and paves the way for a wide range of sports and recreational activities. It can also help to protect and improve health (including health training, compensatory and corrective swimming). Those who are able to maintain rhythmic breathing while swimming have the ability to perform well in emergency situations and, with additional skills, may be able to help others in the water^27^. When the arguments presented here are combined, the swimming skills identified with rhythmic breathing acquire a utilitarian dimension as a tool which may aid the forming of positive attitudes and behaviors in water. Hence, it is justified to give the skill of rhythmic breathing while swimming the attribute of an autonomous water competence^4,6^.

Following the research in the area of the set of essential factors which build water competence^6^—safe water entry^28^; floating and self-orientation skills in and under water^29–32^; swimming skills^4,11,33^; breath control^4,6^; underwater skills^34^; clothed swimming skills^27,35^; also in open water^36^; safe exit from the water^37^; and the ability to recognize and assist a drowning person^8,11^—studies focusing specifically on the need to swim without goggles were not found.

In light of the aforementioned gap in knowledge, the proposal to consider swimming without goggles as an independent water competence will be studied in the context of the ability to maintain rhythmic breathing while swimming with and without goggles. The deficits (mistakes) in hand-breathing coordination and in breath control can lead to life-threatening situations. Therefore, this study is a voice in the discussion about the goals of swimming education—the skills understood as the essential elements of swimming technique (strokes), or the skills giving complete water competence. This is also the voice against the tendency to exaggerate the need of goggles in swimming lessons, because this early and natural discomfort almost never indicates a problem. It is simply a new “skill” to be mastered. It was assumed that prevention or comprehensive elimination of the mistakes in breathing rhythm and swimming without goggles is one the most important pedagogical interventions for solid implementation of water competence (Table 1).

**Table 1:** Assessment scale of crawl swimming skills and classification of breathing control skill used in the study.

Evaluation scale (Points)		1	2	3	4	5
Reliable expert assessment of distance covered (Swimming skills)	Goggles/No goggles	5	10	15	20	25
		Measured in meters				
Reliable expert assessment of breath control skills		4	3	2	1	0
		Measured in the number of mistakes made in the rhythm of breathing				

## Results

**Figure 1:**
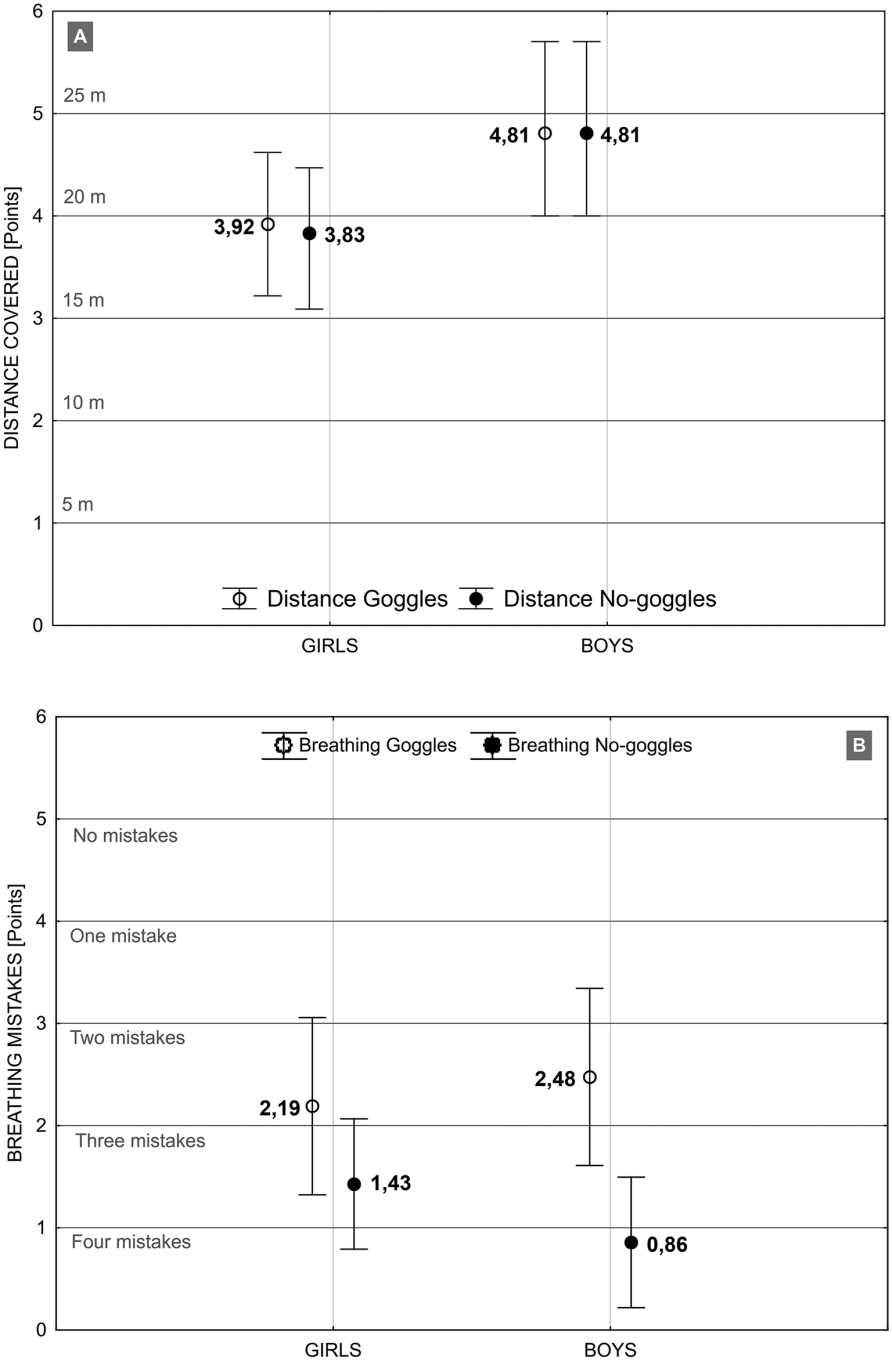
Comparison of mean values obtained by girls and boys in swimming trials with goggles and without goggles in terms of distance covered (**A**) and breathing rhythm mistakes (**B**).

**Figure 2:**
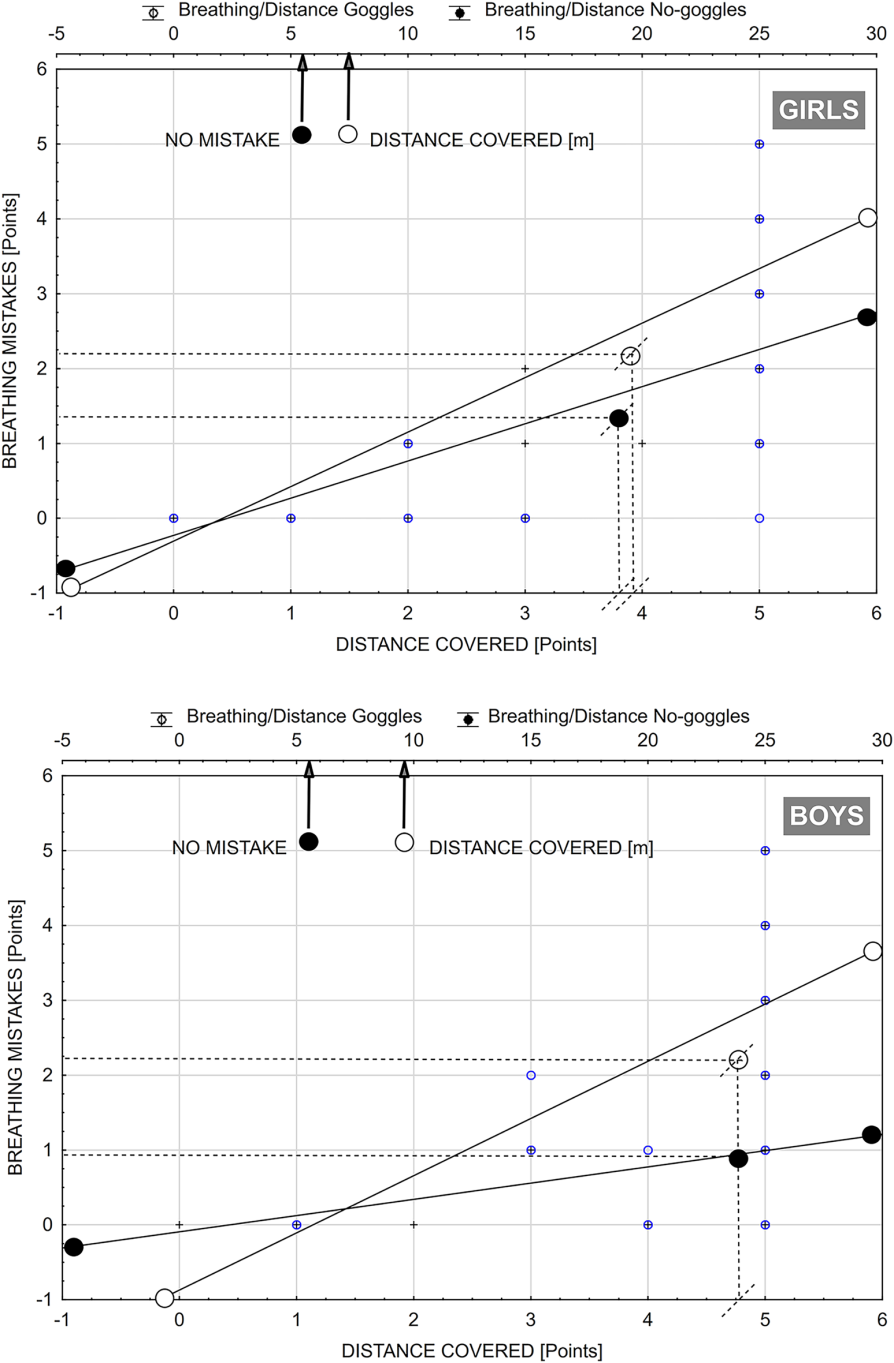
A comparison of the scatter graphs illustrating the number of breathing rhythm mistakes relative to the distance covered by girls and boys when swimming with goggles and without goggles. The mean values and the ability to swim without breathing mistakes is presented above.

The Newman-Keuls post-hoc test (Table 2) did not show evidence of significant differences between the scores of girls and boys, implying that gender does not play a significant role in the scores within the scope of the evaluated aquatic competencies and goggle usage. However, the Student's t-test results for dependent samples, describing statistical significance of the intragroup differences showed, that boys outperformed the girls by 5.7 m with goggles and 4.8 m without goggles (Fig. 1A, Table 3). In both groups fewer breathing mistakes were made when swimming with goggles than without goggles (Fig. 1B, Table 3). The results in Fig. 2 and Table 3 showed that boys made statistically fewer mistakes when swimming with goggles, while girls made fewer mistakes when swimming without goggles. The boys were able to swim with goggles, a statistically significantly longer distance (1.88 m) without breathing mistakes than the girls. In the no goggles trials, the girls swam without breathing mistakes the distance of 2.21 m less than with goggles. Among the boys this distance was 4.28 m shorter than with goggles.

**Table 2:** The Newman-Keuls post-hoc test results describing statistical significance of the intergroup differences between girls and boys in mean values of scores obtained, due to goggle factor.

Evaluated aquatic competencies	Intervention variable	
Swimming distance	Goggles	0.092473
	No goggles	0.070744
Breathing rhythm	Goggles	0.640238
	No goggles	0.208042
Breathing rhythm/Distance	Goggles	0.595136
	No goggles	0.265436

Statistical significance p < 0.05.

**Table 3:** Student's t-test results for dependent samples, describing statistical significance of the intragroup differences in mean values of scores obtained by girls and boys, due to the goggles factor.

Evaluated aquatic competencies	Intervention variable	Girls			Boys		
		Difference in means	t	p	Difference in means	t	p
Swimming distance	Goggles No goggles	0.0953	1.4509	0.162	0.0000	0.0000	1.000
Breathing rhythm	Goggles No goggles	0.7619	3.0735	0.005*	1.7021	4.1651	0.0004*
Breathing rhythm/Distance	Goggles No goggles	0.1866	3.1897	0.004*	0.3425	4.4634	0.0002*

*Statistical significance p < 0.05.

## Discussion

The aim of this study was to examine the ability to maintain the rhythm of breathing in beginning swimmers when swimming with goggles and without. This study concerns the targets of swimming education—the skills understood as the essential elements of swimming, or the abilities giving water competence. It was assumed that comprehensive elimination of the defects in breathing rhythm and swimming without goggles are among the most important elements of pedagogical intervention for solid implementation of water competence.

The girls swam over 19 m, and the boys 24 m in both trials (Fig. 1A). These results are comparable with male physical education students who were able to swim for 25 m^35^. Thus the level of swimming skills of the studied group may be considered as average. The above mentioned, almost five-meter difference in the length of the distance covered (regardless of the swimming pattern), may determine one’s safety in a threatening situation^5^^,^^12^. Although, even poor swimming skills in an emergency situation, when reinforced with high motivation, can provide a better chance for survival^35^.

The interpretation of the results (Fig. 1B) on a 5-point scale of breath control^6^, indicates that the level of breath control of the participants was between the ability to control the spatio-temporal coordination of the arm propulsive movements with free breathing rhythm (Level 2) and the ability to maintain the proper breathing rhythm while swimming in the optimal body position. (Level 3). It is recommended that breath control be emphasized as one of the fundamental skills to be taught, regardless of whether the objective is to learn correct swimming strokes^40^ or to teach natural, safe swimming techniques that do not rely on technical patterns^33^ Assuming, that in a safety-oriented sense, rhythmic breathing confers swimming an efficiency and utilitarian dimension, it should be implemented early in elementary swimming education and continuously improved towards its application when applied to standardized swimming strokes.

In the intragroup analyses, swimming without goggles reduced the efficiency of breath control (Table 2). According to the concept of an enabling constraint Langendorfer^18^, goggles might play a positive role among beginners. This constraint is based mostly on fear of water (or other negative emotional attitudes like skepticism, suspicion, etc.). Because negative emotions regarding water can interfere with activities that take place in or around water, some people avoid the water completely and never learn to swim, while others cannot relax enough to facilitate facial immersion or floating^41^. Additionally, knowing that negative emotions of water are often linked to a previous negative experience^42,43^ it is crucial to avoid creating risky situations regarding goggles within a swimming lesson^44^. In this sense, swimming with goggles is treated as a tool for help to reduce negative aquatic attitudes and behaviors, and then can be used in swimming education. On the contrary, the use of goggles should not be promoted in the safety sense of water competence. Preventing beginners from opening their eyes under water sounds strange here, because the unavailability of goggles in a future emergency situation can reduce the ability to survive. Subsequently, the elimination of goggles from the process of learning to swim is strongly recommended, because with consideration of environmental factors (age, number of people in the group) and on the individual predispositions of the students (level of emotional discomfort, level of progress, motor ability)^19^ it is essential. Using goggles for comfort and pleasure or for performance after obtaining a sufficient level of swimming competence without goggles is clearly allowed, but the point at which goggles may be introduced should be that point at which the learner experiences no advantage to their use^4^ (e.g. regarding breathing)^4^. Given the current discussion, it means clear, that the ability to swim without goggles is one of the crucial, autonomous water competence. To achieve this, it is essential to learn and practice face submersion and basic underwater orientation with bare eyes as a stage of the aquatic literacy process. The link "From Fundamental Swimming Skills to Water Competencies" ^15^ make it clear visible.

The thesis that in the area of maintaining a constant rhythm of breathing at a distance covered, the girls were more accomplished than the boys regardless of the use or non-use of goggles (Tables 3 and 4), may result from the fact that women can visualize differently than men, and contrary to appearances, visual control is important in swimming. This is confirmed by the results of studies of elite swimmers with various visual impairments, which showed that visual acuity was the most evident predictor of total race time^45^.

**Table 4:** The values of Pearson’s correlation coefficients estimated between parameters, evaluated during the trials in girls and boys groups.

Evaluated aquatic competencies	Intervention variable	Distance		Breathing		Breathing/Distance	
		Goggles	No Goggles	Goggles	No Goggles	Goggles	No Goggles
GIRLS n = 25							
Distance Covered	Goggles	X	0.99*	0.77*	0.60*^a^	0.75*	0.60*^a^
	No Goggles	0.99*	X	0.78*	0.62*^a^	0.75*	0.62*^a^
Breathing	Goggles	0.77*	0.78*	X	0.84*^a^	0.99*	0.84*^a^
	No Goggles	0.60*^a^	0.62*^a^	0.84*^a^	X	0.81*^a^	1.00*
Breathing/Distance	Goggles	0.75*	0.75*	0.99*	0.81*	X	0.81*^a^
	No Goggles	0.60*^a^	0.62*^a^	0.84*^a^	1.00*	0.81*	X
BOYS n = 25							
Distance Covered	Goggles	X	1.00*	0.53*	0.29^a^	0.47*	0.21^a^
	No Goggles	1.00*	X	0.53*	0.29^a^	0.47*	0.21^a^
Breathing	Goggles	0.53*	0.53*	X	0.40^a^	0.99*	0.35^a^
	No Goggles	0.29^a^	0.29^a^	0.40^a^	X	0.41^a^	0.98*
Breathing/Distance	Goggles	0.47*	0.47*	0.99*	0.41^a^	X	0.39^a^
	No Goggles	0.21^a^	0.21^a^	0.35^a^	0.98*	0.39^a^	X

*Statistical significance p < 0.05.

^a^The values of Pearson correlation coefficients differentiated the scores obtained in the girls and the boys groups.

The sex differences in the organization of the praxis system in the left hemisphere of the brain have been confirmed by Chipman et al.^46^. There, a system of specialized control of movement—as in swimming—with minimal visual inspection, is located^47^. In women, unlike men, the field of vision did not affect the length of the step when walking^48^. Thus on the analogy of cyclical movements in swimming, as a consequence, men may rely less on visual control in this type of task than women. Paraphrasing Connolly’s^49^ investigation, the perception of action (depending on the use of goggles) and the performance of action maintaining constant coordination of rhythmic breathing, at distance covered, interact bi-directionally, with each other. This suggests that perception and action have bilateral neural mechanisms based on activation of the system of mirror neurons. It is possible that the efficiency of their functions is subject to learning and that a higher level of activation/specialization of the mirror neuron system distinguishes „good swimmers” from beginners. Hence, a stronger justification for the postulate of teaching breath control in swimming with impaired vision conditions (no goggles) appears—also in the sex domain.

Taking into account the multiple levels of the competence of breath control^5^, it seems that the boys studied “can swim” (displace yourself in the water) but do not have ”the competence” to swim effectively/safely—maintaining the rhythm of breathing—regardless of external disturbances (lack of goggles). These findings, in the scope of desirable pedagogical intervention, suggest that educators should consider sex differences in the feedback between visual perception (the goggle factor) and motor control (breath control).

In boys, this mechanism seems to be less efficient than in girls, Therefore, when analyzing the observed mistakes, an attempt was made to identify the relationships between the variables expressed by the factor of goggles usage and the factor of rhythm of breathing—at distance covered (Table 4).

Among girls, both while swimming with goggles and without, a significant relationship was observed between the level of breathing skills (rG = 0.84) and its translation into the distance relatively covered (rG = 0.81). Thus, assuming that in both groups the level of goggle swimming skills paralleled the level of no-goggle swimming skills (rG = 0.99; rB = 1.0), it can be suggested that among girls, goggles usage gives rise to fewer mistakes in maintaining a constant rhythm of breathing at the distance covered. In both groups the level of swimming skills corresponded with the level of breathing skills over the given distance (with goggles rG = 0.75; no-goggles rG = 0.75 and rB = 0.47 and rB = 0.47 respectively) (Table 4). Noting the greater commonality of these relationships among the girls, It also appears that in adolescents with a higher level of swimming skills (regardless of the factor of goggle usage), there were smaller deficits in the area of breath control.

In the case the girls, in trials without goggles, there were significant relationships between the level of swimming skills and the level of breathing skills (with goggles rG = 0.60, rB = 0.29 and no-goggles rG = 0.62, rB = 0.29) and also with breathing at the distance covered without goggles (with goggles (rG = 0.60, rB = 0.21 and without goggles rG = 0.62, rB = 0.21) (Table 4). Among the boys, the lack of relationship between the level of swimming skills and the level of breath control at the distance covered (Table 4). May indicate the source of mistakes in swimming without goggles. It also seems that a higher level of rhythmic breathing skills resulted in fewer mistakes in breath control at distance covered (with goggles rG = 0.99 and rB = 0.98; without goggles respectively rB = 0.99 and rG = 1.0).

However, among the girls it was observed that the higher the level of breathing skills when swimming without goggles, the higher the level of breath control at distance covered with goggles (rG = 0.81) as well as a positive correlation between the level of breathing skills in goggles and the level of breath control at distance covered without goggles (rG = 0.84) (Table 4). These results suggest the girls' adaptability to control breathing at the given distance covered regardless of the factor of goggle usage. Among the boys, there was no such transfer, which may be interpreted as a source of mistakes in the area of breath control competence.

As can be deduced, the feedback between visual perception and motor control suggests that breath control treated as an autonomous component of water competence should defined as meaning swimming without goggles. Starting without goggles (or using them only as a learning aid only in justified cases) could bring the learner to be fully competent in the water without goggles (including breath control). When the goggles are used in the traditional way—as an integral part of the beginners’ swimsuit, it be very difficult to achieve a pedagogical effect that meets the safety criteria. The subjects of this study appeared to perform rhythmic breathing better with googles than without, this appears to demonstrate, that poorer performance without goggles increases risk in any future risk scenario. Such dependence must be avoided at all costs. Additionally not-with-standing the possible positive effects described above, it seems that the necessity from with to without goggles or from without to with goggles, increasing both the time needed for acclimatization to the new condition and for re-acclimatization.

Doubts may be raised about the limitations of conclusions drawn. The first, of them is the size of the sample, but it is indeed comparable to other studies cited here^7,19,25,29,30^. The second one concerns the question, why the front crawl swimming has been assessed. This stroke was chosen because most learners find it the most challenging regarding breath control. Rhythmic breathing requires not only specific skills to coordinate breathing with arm movements (regarding: head position with body rotation and timing of the arm recovery phase affecting breathing time), but also sufficient time to allow consolidation, i.e. to become efficient and eventually reflexive. This way deficits in skills/competence studied have been clearly demonstrated. Future research should consider the question, what would have happened if the participants had started the learning process without goggles?

## Conclusions

In this study, the analysis of rhythmic breathing relative to the use of goggles shows that more mistakes were committed without goggles. Given that goggles were freely used in the prior teaching phase of the participants the suggestion arises that the subjects were “dependent” on goggles. This is dangerous, therefore the recommendation to include aquatic behavior without goggles as an autonomous component of water competence from the beginning of elementary swimming education seems to be the value of this research.

Relative to breath control as an autonomous component of water competence, it is too rarely suggested that this must be without goggles. Consistently with the theory of feedback between visual perception (the goggle factor) and motor control (the breath control factor), it appears that girls, independent of the goggle factor, have a greater adaptive capacity to maintain a continuous breathing rhythm than boys. Therefore, these gender differences should be accepted, especially in the process of elementary swimming education.

It seems that the pedagogical intervention for attaining a stable breathing rhythm without goggles should focus on abilities defined by water competence, rather than on the improvement the skills in technical swimming (strokes). Therefore, the specific training of "free" swimming seems to be crucial for the acquisition of these both water competence.

Adaptation to swimming without goggles right from the very beginning is crucial and should be performed as a part of aquatic literacy process. Here pedagogical patience ((change of teaching perspective) is needed because this process might be elongated in time-line. The use of goggles should be taken into consideration only for those people, for whom the start of the aquatic literacy without goggles is a critical constraint. At an intermediate or advanced phase of learning, careful observation and evaluation must be employed to determine at what point in time it is safe to employ goggles when teaching, without the danger of promoting addiction. The time to introduce goggles is that point when the learner experiences no difference between with and without goggles, especially in relation to rhythmic breathing.

## Methods

### Participants

The participants included 25 boys and 25 girls aged 12–13 years. Irrespective of the level of psychological development they can be situated at the border line between preteens and adolescents—close to puberty^38^. All of them were recruited from three schools of the same district in a city of 800,000 inhabitants. They voluntarily participated as one group in a basic swimming course (thirteen 45-min lessons held once a week. Goggles were allowed during the lessons, but they were not compulsory. Most students used them regularly because the standard curriculum did not include water activities without goggles**.** The learning program led by the same two swimming teachers included the first stage called “water readiness,” as well as back crawl and front crawl swimming. It was assumed that all children participating in the course had similar levels of aquatic experience and knowledge, which was reflected in their individual swimming skills. Although the participants differed from one another, this was not considered to be an issue because each subject controlled and assessed their own swimming skills in the repeated trials. Therefore, the water experience accumulated by each of them before the research session was not assessed. All participants were healthy, and no one reported any temporary indisposition. Their level of fitness on the day of the test appeared to be optimal.

### Experimental design and tasks

The special experimental sessions were organized (2 × 45 min = 90 min). All participants performed the standard warm-up in the water, including five minutes of games and play and five minutes of swimming with the strokes or "semi-strokes" known before. The warm-up did not include any of the elements that would be assessed during the experiment. After 15 min of rest, in the first trial, participants swam in random order as far as possible (up to 25 m) using the front crawl while wearing typical swimming goggles. After a 30-min rest, an identical procedure was conducted with the same participants, but they were asked to swim without goggles. The time interval between individual trials eliminated the impact of fatigue on the quality of their performance.

All trials were performed in a 25-m indoor swimming pool (depth 1.5–1.8 m) with the water temperature maintained at 27 ± 1  C. Evaluation of the swimming skills was based on the distance covered (up to 25 m with an accuracy of 1 m) and the number of mistakes in rhythmic breathing. Performance was evaluated objectively using the Delphi method, modified by Keegan et al., with evaluation by three experts^39^.

The distance covered by each participant was the first criterion of the assessment. The second one was the repeatable rhythm of breathing. Lateral and bi-lateral breathing were allowed in two, three, and four arm movement variants. The head position was not taken into consideration. The end of the trial was defined when the participant stopped swimming (stood on the bottom or grabbed the rope or the edge of the pool). Additionally, the experts paid attention to cyclical coordination of the arm stroke. Recovering the elbows above water with no “dog paddle” was also required. The breathing rhythm in front crawl swimming was chosen because this coordination is widely recognized as the most difficult in all swimming strokes. On the other hand, the children swam at a self-determined pace. The tasks were intentionally arranged to give the participants an opportunity to perform both trials in the simplest, most natural way, which provided optimal conditions for assessment.

Following the aforementioned authors, it was assumed that the distance covered is one of the important indicators of the level of swimming skill. Based on the definition of breath control competence^5,6^, it was assumed that the ability to coordinate in time and space, arm movements, and stable rhythm of breathing without stopping is an indicator of breath control while swimming the crawl stroke. The ability to keep the breathing rhythm during swimming measured by a number of mistakes in breathing rhythm relative to the distance covered was treated as a measure of breath control competence.

A summary of the trials and the assessment scale are presented in Table 1, The scoring scale for both aquatic skills assessed (in both intervention variables) was the same—from one (minimum) to five (maximum) points. For distance covered (swimming skills) it increased 1 point every 5 m up to 25 m (5 points). The number of mistakes made in the breathing rhythm during the distance covered was used to assess of breathing control. The value of the points decreased with each mistake—from zero (5 points) up to 4 mistakes (1 point).

### Statistical analysis

Statistical analyses were performed using Statistica 13.0 software (StatSoft, Tulsa, OK, USA). All variables fulfilled the prerequisites of the parametric test for normally distributed data, which was assessed using the Shapiro–Wilk test. The homogeneity of variance was also confirmed by Levene’s variance ratio test. Paired t-tests, performed separately for the group of girls (n = 25) and boys (n = 25), were used to compare the differences in assessment of front crawl swimming skills performed with and without goggles. The same statistical tool was used to compare the differences in self-assessment of these adolescents before and after the previously mentioned trials. One-way ANOVA (based on gender) followed by the Newman-Keuls post hoc test was used for intergroup analysis. Pearson’s correlation coefficients were computed to assess the level of association (p ≤ 0.05) between pairs of variables. Correlation effect sizes were deemed as: weak, 0 <|R|≤ 0.1, small, 0.1 <|R|≤ 0.3, moderate, 0.3 <|R|≤ 0.5, or strong, |R|> 0.5.

### Ethics approval

All methods and procedures performed in this study were in accordance with the relevant guidelines and regulations, reviewed and approved by the local ethics committee (Ethical Committee of the University School of Physical Education in Wroclaw, Poland—aproval number 126/2018) and with the 1964 Declaration of Helsinki and its later amendments. The juvenile participants and their parents or caregivers were informed about the objectives and procedures of the experiment. Prior to participation, a written informed consent was signed by parents/caregivers of the participants.

## Data Availability

The datasets generated during and analysed during the current study are available from the corresponding author on reasonable request.
